# Bacteraemia due to *Microbacterium paraoxydans* in a patient with chronic kidney disease, refractory hypertension and sarcoidosis

**DOI:** 10.1099/jmmcr.0.005169

**Published:** 2018-10-31

**Authors:** Matthew S. Chorost, Nancy C. Smith, Jack N. Hutter, Ann C. Ong, Jason A. Stam, Patrick T. McGann, Mary K. Hinkle, Kurt E. Schaecher, Edwin Kamau

**Affiliations:** ^1^​ Walter Reed National Military Medical Center, Bethesda, MD, USA; ^2^​ Walter Reed Army Institute of Research, Silver Spring, MD, USA

**Keywords:** *Microbacterium paraoxydans* infection, erythema, tenderness and discharge, dizzy spells, IV vancomycin, PO levofloxacin

## Abstract

**Introduction:**

*Microbacterium* spp. are yellow-pigmented Gram-positive coryneform rods found in various environmental sources, such as soil and water samples. They rarely cause human infection, mostly infecting immunocompromised patients and catheter insertion sites, making them challenging to identify in clinical settings.

**Case presentation:**

We report a case of a 61-year-old female on long-term prednisone therapy for sarcoidosis with minimal exposure to environmental sources, who presented with an overtly infected Hickman catheter site and presyncope. The patient had a central venous catheter (CVC) that had been in place for the previous 6 years for treatment of refractory hypertension and congestive heart failure. Blood cultures obtained from the CVC on initial presentation were positive for a mixed infection, which was subcultured and grew *Staphylococcus aureus*, *Staphylococcus epidermidis*, *Acinetobacter radioresistens* and *Leifsonia aquatica* based on the Becton Dickinson Phoenix Automated Microbiology System. The *L. aquatica*, designated as isolate 4120, was further analysed, since infections associated with this organism are uncommon, and it was the only organism to grow from the patient’s catheter tip. Matrix-assisted laser desorption ionization–time of flight MS identified isolate 4120 as *Microbacterium paraoxydans*. To resolve the conflicting results, additional analyses of isolate 4120 were carried out and compared to several reference strains. Isolate 4120 was found to have intermediate susceptibility to ciprofloxacin and non-susceptibility to vancomycin. Morphology, susceptibility, biochemical characteristics and whole-genome sequencing confirmed the clinical isolate as *Microbacterium paraoxydans*.

**Conclusion:**

In this case, we identified an organism that is rarely seen in clinical settings and characterized it with a comprehensive laboratory analysis. The patient in our case responded to replacement of the CVC, and treatment with levofloxacin by mouth and intravenous vancomycin.

## Introduction

*Microbacterium* species are yellow-pigmented Gram-positive coryneform rods isolated from various environmental sources, such as soil, water, sewage, hospital humidifiers and hospital air [1–3]. The genus *Microbacterium*, which was redefined from the genera *Microbacterium* and *Aureobacterium*, has 104 different species to date (List of Prokaryotic Names with Standing in Nomenclature, www.bacterio.cict.fr/m/microbacterium.html, accessed February 12, 2018). Human infections with *Microbacterium* species are uncommon and typically associated with immunocompromised patients or indwelling catheters [4].

*Microbacterium* are rarely encountered in clinical settings, but when present, can pose challenges in identification [2, 5]. This has likely resulted in an incomplete picture of the bacterium’s epidemiology, optimal treatment and prognosis [2]. The coryneform bacteria are usually identified based on their morphological and biochemical profiles, in which a series of biochemical reactions are interpreted with a database or with automated systems such as the Becton Dickinson (BD) Phoenix Automated Microbiology System. Unfortunately, the discriminating power of the biochemical reactions has limitations. The 16S rRNA gene is useful for identification and confirmation as a ‘gold standard’ for many bacteria including *Microbacterium* species, because it is highly conserved within and among species of the same genus [2]. More recently, matrix-assisted laser desorption ionization–time of flight mass spectrometry (MALDI-TOF MS) has played an increasing role in clinical microbial identification. In this report, we present a case of bacteraemia associated with a long-term central venous catheter (CVC) due to *Microbacterium paraoxydans*, a *Microbacterium* species that is rarely encountered in human infections [3–6].

## Case report

In July 2016, a 61-year-old female suffering from chronic kidney disease, secondary to refractory hypertension, on long-term treatment with 15 mg prednisone for sarcoidosis, presented to the emergency room with presyncope, and drainage and erythema at her Hickman catheter insertion site. She had had the CVC in place for the previous 6 years for treatment of frequent episodes of malignant hypertension and congestive heart failure requiring urgent administration of antihypertensive in the setting of poor venous access. Five days prior to admission, the patient noticed that the catheter site had become erythematous and tender with copious brown discharge, which required her to change dressings daily rather than weekly. She had been experiencing presyncopal spells since the discharge started. Three days prior to admission, she started taking 250 mg each day of unused, unexpired oral levofloxacin she had left over from a prior urinary tract infection. This led to an initial decrease in erythema, tenderness and discharge with resolution of the presyncopal spells. After three days, the presyncopal spells returned with nausea, which lead her to seek treatment. In the emergency room, she had vitals within the normal range. She denied having any other symptoms, but reported that she had been showering with the catheter uncovered for over a year. Aerobic and anaerobic blood culture bottles were set up (BD BACTEC blood culture media) from the CVC, which was removed to eliminate the most likely source of infection. On admission, the patient had a total white cell count of 1.04×10^4^ cells µl^−1^, a haemoglobin value of 12.5 g dl^−1^ and a platelet count of 3.55×10^5^ platelets µl^−1^. The erythrocyte sedimentation rate was 33 mm h^−1^ and the C-reactive protein level was 2.1 mg dl^−1^. Daily blood cultures were obtained from peripheral sites over the next 3 days, which were negative. On admission, the patient was placed on 1000 mg of intravenous vancomycin every 12 hours and the oral levofloxacin was increased to 750 mg each day to cover polymicrobial infections of the catheter. The patient responded well to treatment after completing a 14 day course of therapy with a peripherally inserted central catheter (PICC) line that was placed on the third day of admission. Two months after admission, she had a port placed for permanent access with a lower risk of infection, and had not had evidence of infection since the port’s placement.

**Fig. 1.**
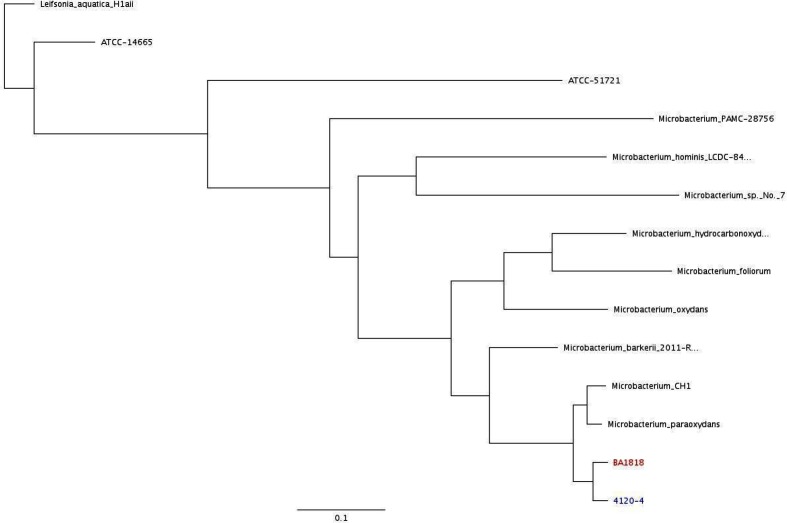
Core-genome-based phylogeny tree showing the relationship of the patient isolate to related species. The tree was inferred from whole-genome sequence data from this study for the patient isolate (4120) and three reference strains [ATCC 1818 (*Microbacterium paraoxydans*), ATCC 14665 (*L. aquatica*) and ATCC 51721 (*L. aquatica*)]. The rest of the sequence data was obtained from the National Center for Biotechnology Information. Names and accession numbers are given as cited in the GenBank database.

The aerobic and anaerobic blood bottles initially collected from the central line before the patient was admitted grew Gram-positive cocci in clusters, Gram-negative rods and Gram-positive rods in 24 h. Both aerobic and anaerobic blood bottles were subcultured on 5 % sheep blood agar and chocolate agar plates (Remel) per the standard protocol. The initial identification and susceptibility testing of colonies from the plate media was carried out using the BD Phoenix. The bacteria were identified as *Staphylococcus aureus*, *Staphylococcus epidermidis*, *Acinetobacter* sp. and *Leifsonia aquatica* (score of 90 %). The Hickman catheter tip grew small white and small yellow colonies that matched the colonies from the blood cultures identified as *L. aquatica*. The *L. aquatica*, designated as isolate 4120, was further analysed, since infections associated with this bacterium are uncommon. MALDI-TOF MS was used for microbial identification, following the manufacturer’s instructions. Isolate 4120 was identified as *Microbacterium paraoxydans* with a score of 2.13. The MALDI-TOF MS BioTyper 3.0 software queries a reference database and returns top organisms with confidence scores ranging from 0.0 to 3.0. Scores of ≥2.0 are considered high-confidence with identification to the species level, while scores of 1.6 to 1.99 are considered intermediate-confidence with identification to the genus level only. Scores of <1.6 are considered unacceptable for identification, according to manufacturer’s recommendation [7]. Due to the conflicting results between the BD Phoenix and the MALDI-TOF MS, the RapID CB PLUS system (Thermo Scientific) was used to further investigate the identity of this isolate. The identification came back as *L. aquatica* with a score of >99 %. This prompted us to do additional investigation and analyses. Reference strains of *L. aquatica* (ATCC 14665 and ATCC 51721) and *Microbacterium* sp. (*Microbacterium paraoxydans* ATCC 1818, *Microbacterium foliorum* DSM-12966 and *Microbacterium oxydans* DSM-20578) were obtained from the American Type Culture Collection (Manassas, VA, USA) or the DSMZ (Leibniz Institute DSMZ, Brunswick, Germany), respectively. Morphological and biochemical profiles were established using standard methods. The susceptibility testing of colonies from the plate media to MICs was carried out using the BD Phoenix system, confirmed on the Trek Sensititre automated testing platform, bioMérieux Etest strips and/or Kirby–Bauer antibiotic, following the manufacturers’ instructions. Results were interpreted as per Clinical and Laboratory Standards Institute (CLSI) guidelines [8]. Isolate 4120 and three of the reference strains (ATCC 1818, ATCC 14665 and ATCC 51721) underwent whole-genome sequencing using the MiSeq Genetic Analyzer (Illumina). The morphological, biochemical and susceptibility characteristics of isolate 4120 were most similar to those of ATCC 1818, which is *Microbacterium paraoxydans* (Table 1). Notably, isolate 4120 was highly resistant to erythromycin and rifampicin. The MIC for vancomycin was 3 µg ml^−1^, which is considered non-susceptible. The score-oriented dendrogram generated by the BioTyper software showed the MALDI-TOF MS mass spectra of isolate 4120 cell extracts to be phylogenetically related to *Microbacterium paraoxydans* (data not shown). Further, genetic analysis confirmed that isolate 4120 was most closely related to *Microbacterium paraoxydans* [average nucleotide identity (ANI) 94.01 %] and *Microbacterium* spp. CH1 (ANI 93.97 %) based on the sequences available in GenBank. Notably, the 4120 genome shared an ANI score of >97.7 % with *Microbacterium paraoxydans* ATCC 1818 (Fig. 1). Based on this data, we conclusively identified isolate 4120 as *Microbacterium paraoxydans*. Interestingly, with the exception of one reference strain (ATCC 14665, *L. aquatica*), the BD Phoenix and RapID CB PLUS systems incorrectly identified all organisms that we attempted to identify (Table 2). Of note, the BD Phoenix identified ATCC 14 665 as *L. aquatica* in the first run with a score of 99 %, but on the second run, the organism was identified as *Cellulomonas turbata* with a score of 99 %. Similarly, the system also identified the test isolate (4120) as *L. aquatica* in the first run with a 90 % score, but in the second run, it was identified as *Micrococcus lylae* with a score of 98 %. The MALDI-TOF MS only failed to identify (score was too low) reference strain ATCC 51721, which also could not be readily classified based on DNA sequence (Fig. 1).

**Table 1.** Antimicrobial-susceptibility patterns of the test isolate and reference strains The antimicrobial-susceptibility values shown are the MICs in µg ml^−1^ for the test isolate (4120) and the reference strains. MIC interpretive criteria were based on the CLSI M45-ED3 document [8], table 6. The interpretive criteria are shown in brackets (susceptible, S; intermediate, I; resistant, R) for each agent and isolate tested, where applicable. For vancomycin, the interpretive criterium available is for S only, which is set at ≤2. The CLSI states that the absence or rare occurrence of resistant strains precludes defining any results categories other than S.

**Antibiotic**	**4120 (test isolate)**	**ATCC 1818 (*Microbacterium paraoxydans*)**	**ATCC 14 665 (*L. aquatica*)**	**ATCC 51 721 (*L. aquatica*)**	**DSM-12 966 (*Microbacterium foliorum*)**	**DSM-20 578 (*Microbacterium oxydans*)**
Ampicillin	0.5	0.75	32	0.75	0	2
Penicillin	0.75 (I)	1 (I)	8 (R)	0.38 (I)	0 (S)	1.5 (I)
Ciprofloxacin	1.5 (I)	2 (I)	1.5 (I)	0.125 (S)	0 (S)	1.5 (I)
Clindamycin	4 (R)	3 (I)	6 (R)	48 (R)	0 (S)	1 (I)
Erythromycin	32 (R)	0.064 (S)	0.016 (S)	>256 (R)	0 (S)	1.5 (I)
Gentamicin	2 (S)	2 (S)	0.5 (S)	>256 (R)	0 (S)	0.75 (S)
Rifampicin	>32 (R)	4 (R)	0.125 (S)	0.003 (S)	0 (S)	1 (S)
Tetracycline	1 (S)	1.5 (S)	0.75 (S)	0.13 (S)	0 (S)	4 (S)
Vancomycin	3	3	12	0.5 (S)	0 (S)	4

**Table 2.** Identification of bacteria using the different methods Each identification method was run in replicates where bacteria were prepared on different days and then analysed. Data shown here is from two runs (replicates). For BD Phoenix and RAPID CB, the ID or per cent identity from the replicate runs is shown, if different. For the MALDI-TOF MS, the organism ID and the score are shown for each run. Based on sequence analysis, 51 721 is *Microbacterium* spp. but additional analysis is needed; see Fig. 1.

**Organism ID**	**BD Phoenix**	**MALDI-TOF MS**	**RAPID CB**	**Sequence ID**
4120 (test isolate)	*L. aquatica* 90 %/*Micrococcus* lylae 98 %	*Microbacterium paraoxydans* 2.13	No ID	*Microbacterium paraoxydans*
ATCC 1818 (*Microbacterium paraoxydans*)	*Dermacoccus nishinomiyaensis* 98 %/90 %	*Microbacterium paraoxydans* 2.08	*L. aquatica* >99.9 %	*Microbacterium paraoxydans*
ATCC 14 665 (*L. aquatica*)	*L. aquatica* 99 %/*C.* *turbata* 99 %	*L. aquatica* 2.25	*L. aquatica* >99.9 %	*L. aquatica*
ATCC 51 721 (*L. aquatica*)	*Kytococcus sedentarius* 97 %/97 %	*Lactobacillus kalixensis* 1.51/*Cupriavidus* *necator* 1.33	*Brevibacterium casei* >99.9 %	?
DSM-12 966 (*Microbacterium foliorum*)	*C. turbata* 99 %/95 %	*Microbacterium foliorum* 2.54	*Oerskovia* spp. >99.9 %/no ID	Not sequenced
DSM-20 578 (*Microbacterium oxydans*)	*L. aquatica* 99 %/99 %	*Microbacterium oxydans* 2.47	*L. aquatica* >99.9 %	Not sequenced

## Discussion

In this report, we have identified and described a case of *Microbacterium paraoxydans* associated with CVC infection. Infections caused by *Microbacterium* spp. are rare, present diagnostic challenges and most cases occur in oncology patients [9, 10]. *Microbacterium paraoxydans* was first described as a new species in Belgium, collected in a patient in 1997 (reported in 2003) with leukemia and CVC-associated bacteraemia [4]. The second case of *Microbacterium paraoxydans* bacteraemia was reported in 2011 in a patient on parenteral nutrition for an unknown enteropathy with no significant immunodeficiency [5]. *Microbacterium paraoxydans* has also been associated with peritonitis from peritoneal dialysis catheters in two recent cases, for which exposure to agriculture environmental factors seemed to be an important risk-factor [3, 6]. One of the strongest risk factor associated with *Microbacterium* spp. (including *Microbacterium paraoxydans*) infection in both immunocompromised and non-immunocompromised patients is the presence of long-term catheters, such as chemo-ports or haemo-dialysis catheters [2–5, 9–11], which also appears to be a risk factor in our case.

*Microbacterium paraoxydans* has also been found in archived clinical isolates, which were initially characterized as *Microbacterium* spp. Of 30 archived *Microbacterium* species isolates from a hospital in Belgium, the majority were either *Microbacterium oxydans* (9 of 30) or *Microbacterium paraoxydans* (5 of 30) [4]. In 2008, Gneiding *et al*. characterized 50 archived *Microbacterium* clinical strains collected over a 5 year period in a hospital in Germany. With 18 different taxa encountered, the majority were *Microbacterium oxydans (n=11)*, *Microbacterium paraoxydans* (*n*=9) and *Microbacterium foliorum* (*n*=7) [12]. The specimens were primarily obtained from adults from a variety of clinical sources, including a wound, urine, blood, tracheal secretions and other sites.

The difficulty in the identification of coryneform bacterial strains to the genus level using biochemical methods in most clinical laboratories have been described, and even when isolated from clinical specimens, they are often considered to be contaminants [2]. The API Coryne system is considered unreliable for the diagnosis of pigmented Gram-positive bacilli [2, 10, 12, 13]. In our study, both the biochemical methods using the BD Phoenix and RapID CB PLUS system wrongly identified the patient isolate as *L. aquatica*, both with a high score. The BD Phoenix is an automated system, which identifies a broad range of Gram-positive and Gram-negative bacteria using modified conventional, fluorogenic and chromogenic substrates. In contrast, the RapID CB Plus system is a manual system composed of four rapid carbohydrate utilization tests and 14 single-substrate enzymatic tests [14]. MALDI-TOF MS is now widely used in laboratories worldwide in clinical microbiology settings, because it is a relatively inexpensive, rapid and accurate method for the identification of cultured bacteria and fungi based on automated analysis of the mass distribution of bacterial proteins [7]. As of 2017, Bruker’s MALDI BioTyper has an Food and Drug Administration cleared reference library of more than 400 microorganisms, making it highly reliable system for identification of rare infections. In addition to providing the correct identification of the clinical isolate from this report, MALDI-TOF MS provided accurate identification of the reference strains that were analysed, with the exception of *L. aquatica* (ATCC 51721), whose identification could not be verified with whole-genome sequencing either. This reference strain (ATCC 51721) will require additional analysis to confirm its identity. This study shows that in conjunction with sequencing, MALDI-TOF MS can be a reliable system for the identification of rarely encountered organisms such as coryneform rods that might otherwise be difficult to identify.

With the exception of erythromycin and rifampicin, the susceptibility profile of the patient isolate 4120 was most similar to that of the reference strains *Microbacterium paraoxydans* (ATCC 1818) and *Microbacterium oxydans* (DSM-20578). The sensitivity profile of isolate 4120 shows this bacteria had intermediate sensitivity to ciprofloxacin and was non-susceptible to vancomycin. Despite the isolate being non-susceptible, our patient responded well to vancomycin and levofloxacin treatment, which was targeted for the most pathogenic agent, *S. aureus* and the *Acinetobacter*. This suggests removal of the infected catheter may have been a useful intervention to successfully treat a *Microbacterium paraoxydans* infection.

Our case report has provided an opportunity to review the available literature related to *Microbacterium paraoxydans* infections. The organism can prove challenging to diagnose by traditional microbiological methods, but is readily identifiable with MALDI-TOF MS. Infections due to *Microbacterium paraoxydans* are likely to be associated with long-term catheters and environmental factors, and might have unpredictable antibiotic sensitivities, but the removal of the catheter is critical for the treatment.

## References

[1] Funke G, Falsen E, Barreau C (1995). Primary identification of *Microbacterium* spp. encountered in clinical specimens as CDC coryneform group A-4 and A-5 bacteria. J Clin Microbiol.

[2] Lau SK, Woo PC, Woo GK, Yuen KY (2002). Catheter-related *Microbacterium* bacteremia identified by 16S rRNA gene sequencing. J Clin Microbiol.

[3] Choi HS, Bae EH, Ma SK, Kim SW (2017). Peritoneal dialysis-related peritonitis caused by *Microbacterium paraoxydans*. Jpn J Infect Dis.

[4] Laffineur K, Avesani V, Cornu G, Charlier J, Janssens M (2003). Bacteremia due to a novel *Microbacterium* species in a patient with leukemia and description of *Microbacterium paraoxydans* sp. nov. J Clin Microbiol.

[5] Enoch DA, Richardson MP, Hill RL, Scorer PM, Sismey A (2011). Central venous catheter-related bacteraemia due to *Microbacterium paraoxydans* in a patient with no significant immunodeficiency. J Clin Pathol.

[6] Miyamoto M, Sakurada T, Oishi D, Koitabashi K, Hanada K (2013). The first case report of peritoneal dialysis related peritonitis caused by *Microbacterium paraoxydans*. Clin Nephrol.

[7] Patel R (2015). MALDI-TOF MS for the diagnosis of infectious diseases. Clin Chem.

[8] CLSI (2015). Performance Standards for Antimicrobial Susceptibility Testing.

[9] Adderson EE, Boudreaux JW, Hayden RT (2008). Infections caused by coryneform bacteria in pediatric oncology patients. Pediatr Infect Dis J.

[10] Alonso-Echanove J, Shah SS, Valenti AJ, Dirrigl SN, Carson LA (2001). Nosocomial outbreak of *Microbacterium* species bacteremia among cancer patients. J Infect Dis.

[11] Yusuf E, Wybo I, François K, Pipeleers L, Echahidi F (2016). *Microbacterium* spp. as a cause of peritoneal-dialysis-related peritonitis in two patients. J Microbiol Immunol Infect.

[12] Gneiding K, Frodl R, Funke G (2008). Identities of *Microbacterium* spp. encountered in human clinical specimens. J Clin Microbiol.

[13] Almuzara MN, de Mier C, Rodríguez CR, Famiglietti AM, Vay CA (2006). [Evaluation of API Coryne System, version 2.0, for diphteroid gram-positive rods identification with clinical relevance]. Rev Argent Microbiol.

[14] Hudspeth MK, Hunt Gerardo S, Citron DM, Goldstein EJ (1998). Evaluation of the RapID CB Plus system for identification of *Corynebacterium* species and other gram-positive rods. J Clin Microbiol.

